# Supplementary figures and images for: PHACTR1 is associated with disease progression in Chinese Moyamoya disease
Source: PeerJ. 2020 May 5;8:e8841. doi: 10.7717/peerj.8841 (PMC7207206; doi:10.7717/peerj.8841)

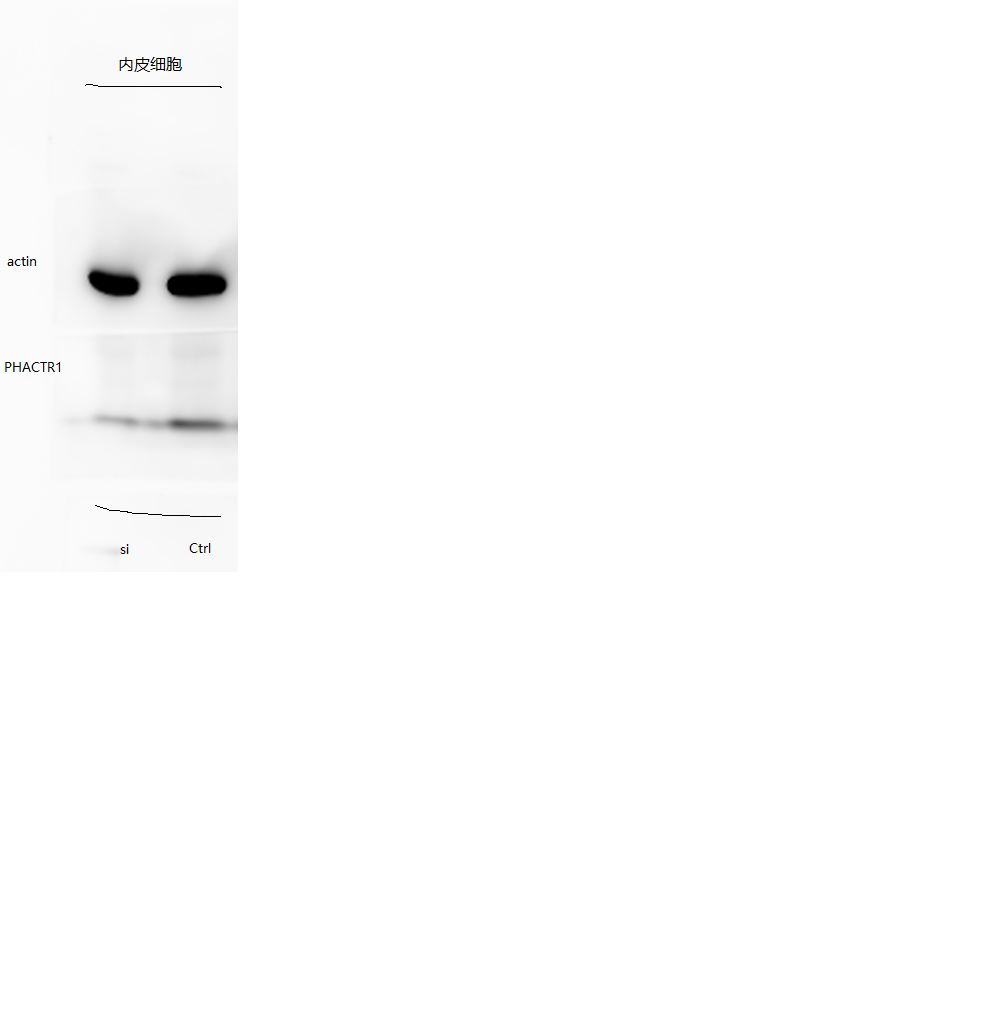

Supplement: Figure S1 [file peerj-08-8841-s001.png]
